# Determinants of Compliance Behaviours among Patients Undergoing Hemodialysis in Malaysia

**DOI:** 10.1371/journal.pone.0041362

**Published:** 2012-08-03

**Authors:** Yoke Mun Chan, Mohd Shariff Zalilah, Sing Ziunn Hii

**Affiliations:** 1 Institute of Gerontology, University Putra Malaysia, Serdang, Malaysia; 2 Department of Nutrition and Dietetics, Faculty of Medicine and Health Sciences, University Putra Malaysia, Serdang, Malaysia; 3 Viva Life Science Private Limited, Petaling Jaya, Malaysia; University of Sao Paulo Medical School, Brazil

## Abstract

**Background:**

Patients with end stage renal disease often fail to follow prescribed dietary and fluid regimen, leading to undesirable outcomes. This study aimed to examine and identify factors influencing dietary, fluid, medication and dialysis compliance behaviours in patients undergoing hemodialysis.

**Methods:**

This was a cross-sectional study which employed purposive sampling design. A total of 188 respondents were recruited from 14 dialysis centres in Malaysia between 2008–2011. Self-reported compliance behaviours and biochemical measurements were used as evaluation tools.

**Results:**

Compliance rates of dietary, fluid, medication and dialysis were 27.7%, 24.5%, 66.5% and 91.0%, respectively. Younger, male, working patients and those with longer duration on hemodialysis were found more likely to be non-compliant. Lacks of adequate knowledge, inadequate self-efficacy skills, forgetfulness and financial constraints were the major perceived barriers towards better compliance to fluid, dietary, medication and dialysis, respectively.

**Conclusions:**

Healthcare professionals should recognise the factors hindering compliance from the patients' perspective while assisting them with appropriate skills in making necessary changes possible.

## Introduction

In Malaysia, there is consistent increase in the incidence of newly-diagnosed individuals with end-stage renal disease (ESRD) which requires renal replacement therapy each year, fuelled by the expansion of the aged population as well as the rapid emergence of diabetic nephropathy [1]. While peritoneal dialysis is the preferred treatment modality in Hong Kong [2] and Mexico [3], hemodialysis is still the predominant mode of treatment for ESRD patients in most countries [4]–[5] including Malaysia [1]. Peritoneal dialysis is grossly underutilized in Malaysia despite the conscious effort by the government to promote peritoneal dialysis as the dialysis modality of choice. This is largely attributed to the readily available hemodialysis centres provided by non-government organisations (NGOs) and private sectors besides better survival rate for hemodialysis patients [1]. Successful hemodialysis is highly dependent on the lifetime commitment of patients to four aspects of regimens, namely dietary guidelines, fluid restriction, medication and dialysis [6]. Although compliance to hemodialysis regimens is critical in the management of hemodialysis patients as failure to do so has been associated with increased risk of medical complications including higher risk of cardiac disease [7], poorer quality of life and decreased life expectancy [8]–[9], nonadherence to one or more aspects of hemodialysis treatment regimen has been widely reported [10]–[11].

The reported prevalence of non-compliance rates among hemodialysis patients varies widely, ranging from 30–74%, 2–81%, 17–46% and 0–32% for compliance to fluid restrictions [12]–[14], diet restrictions [11]–[12], [15], medication [14], [16]–[17] and dialysis [17]–[19], respectively. These variations were partly attributed to the different population being studied and most likely the inconsistency in the measures used to define compliance rates. A number of variables have been identified to influence compliance rate among hemodialysis patients, with varying degrees of agreement between different studies. More consistent reported demographic correlates of non compliant were younger age [15], [17], [20] and male patients [12], [15], [21]. Other variables include education [15], [22], employment status [22]–[23], duration on dialysis [24]–[26], health locus of control and social support [27].

In view of the rapidly increased of ESRD in Malaysia, there is a need to determine the compliance rate to therapeutic regimen among patients undergoing hemodialysis. Previous study identified non-compliance to fluid intake was prevalent among patients undergoing hemodialysis in a single centre in Malaysia [28]. Data on compliance to other treatment regimes (dietary, medication and dialysis attendance) is however not available. Hence this present study aimed to determine the overall compliance behaviour to therapeutic regimens among patients undergoing hemodialysis and to determine the factors contributing to compliance among these subjects.

## Materials and Methods

This was a cross-sectional study with respondents recruited from 14 hemodialysis centres in Malaysia. A total of 346 subjects were screened, 217 were found meeting the selection criteria while eventually 188 respondents consented to participate, giving a response rate of 87%. The study employed purposive sampling as the selection of respondents was based on several eligible criteria. The inclusion criteria entailed receiving hemodialysis for four hour thrice weekly; attended routine hemodialysis treatment for a minimum of three months prior to the study; at least 18 years of age, suffers from no major acute diseases or major psychological disorders. The study protocol was approved by the Medical Research Ethics Committee of Faculty of Medicine and Health Sciences, Universiti Putra Malaysia, in accordance with current guidelines on Good Clinical Practice and the Declaration of Helsinki. Following ethics review board approval, the researchers explained the study to potential participants. Anonymity and confidentially were assured before signed individual consents were obtained from all subjects.

A set of structured questionnaire was developed to ascertain information on patients' demographic characteristics. Treatment conditions, medical history and proxy clinical measures include mean serum potassium and phosphorus levels for the last three measurements were retrospectively obtained from medical record. The modified Charlson's Comorbidity Index which has been validated in dialysis patients [29] was used to quantify subject's comorbidity score. Mean interdialytic weight gain (IDWG) which is defined as weight gain between two consecutive dialysis sessions for the past three months was obtained from subject's dialysis record. A 25-item dietary knowledge questionnaire, which was modified from Durose et al. (2004) [11], was used to assess subjects' knowledge on diet and fluid regimen including food sources for nutrients (e.g., chocolate is good food source for phosphorus), recommended dietary preparation/restriction (e.g., dark green leafy vegetables should be cut small before washing and immerse in water), and possible consequences of noncompliance to dietary recommendation (e.g., excessive intake of sodium and fluid is harmful to the heart). The answers were prepared in yes or no format with an additional “don't know” category to avoid bias attributable to guessing. Each correct response was given a score of one while zero score was given to incorrect or “don't know” response. The scores were weighted and converted to standardized normal distribution, giving a maximum score of 100 for the knowledge scale. The internal consistency (Cronbach's alpha) was 0.8, denoting a reasonable internal consistency of this instrument. A combination of objective and subjective measures was used to access the compliance rates in order to increase the reliability and validity of the compliance results [6].

### Objective measures

Interdialytic weight gain (IDWG), serum potassium and phosphorus which have been widely used in many studies [12]–[13], [30] were used as indicators of fluid and dietary compliance. In view of the absence of validated international cut-off values, the existing acceptable limits used in the dialysis units were applied to identify non-adherers. Subjects were considered as dietary compliant when both serum potassium and phosphorus were within the acceptable ranges. Fluid compliant of subject was defined when mean IDWG for the past three months were within the acceptable range. Predialysis serum phosphate was selected as compliance indicator for medication [14], [22]. Dialysis compliance was determined as the number of appointment or dialysis session skipped compared to the prescribed sessions in a given duration. The data was gathered retrospectively from the subjects' dialysis record books. Attendance to dialysis was classified arbitrarily as non-compliant if subjects skipped at least one dialysis treatment in the month before enrolment into the study.

### Subjective Measures

To evaluate patients' compliance behaviour, a modified dialysis diet and fluid non-adherence questionnaire (DDFQ) [31] was used. There were eight subscales: two each (frequency and intensity) to measure the patients' compliance behaviour to dietary guidelines, fluid, prescribed phosphate binding medication and hemodialysis treatment, respectively. The frequency of non-compliance was assessed for the last 14 days while intensity of non-compliance was evaluated on a 5-point rating scale, where responses ranged from 0 as “very severe deviation” to 4 as “no deviation”.

### Data Analysis

Analyses were performed using the SPSS Windows Version 18 (Chicago, IL). Data were presented as mean standard deviation for continuous variables and percentages for categorical variables. Pearson's product moment correlation coefficients were computed to determine the associations between continuous variables. Stepwise multivariate linear regression analysis was performed to identify variables that predict the compliance indicators. Statistical significance was defined at *p*<0.05.

## Results

Subjects' characteristics are presented in Table 1. The mean age of subjects was 58 years old. There were 48.9% male and 51.1% female. A majority were married (80.3%) with more than half (51.1%) had at least completed secondary education. Approximately three-quarters were either retired or unemployed. Diabetes mellitus and glomerulonephritis were the two major known etiology of renal failure. The presence of co-morbidities was common in this sample, with hypertension being the most prevalent (67.0%) and followed by diabetes mellitus (46.3%).

**Table 1:** Demographics and clinical characteristics of subjects.

Characteristics		Mean (SD)	Range	%
Age (years)		58.2 (10.5)	23 – 75	
Sex	Male			48.9
	Female			51.1
Marital status	Single			12.8
	Married			80.3
	Divorced/Widowed			6.9
Education	No formal education			10.6
	Primary school			38.3
	Secondary school and above			51.1
Employment	Employed			25.5
	Unemployed			66.0
	Retired			8.5
Primary diagnosis of renal failure	Diabetes mellitus			25.5
	Glomerulonephritis			8.5
	Unknown cause			29.8
	Others			37.1
Duration of dialysis (months)		63.2 (39.3)	5 – 162	
Presence of co-morbidity	Hypertension			67.0
	Diabetes mellitus			46.3
	Ischaemic heart disease			14.9
Dry weight (kg)		56.8 (14.0)		
Interdialytic weight gain (kg)		2.8 (0.7)		

As depicted in Table 2, while a total of 48.4% and 36.2% of the subjects perceived themselves as fluid or dietary compliant, approximately one-quarter of the subjects were actually adhered to dietary (27.7%) and fluid (24.5%) restrictions. Based on self-reported data, 16 subjects missed at least one dialysis session while the dialysis record book indicated 17 subjects actually skipped at least one dialysis session. This gave a high consistency in dialysis compliance rates between self-reported data and data retrieved from patients' dialysis record. On the other hand, self-reported compliance to medication was 50.5%, while clinically determined rate using serum phosphorus was 66.5%. The percentage of self-reported non-compliance (mild to very severe) to prescribed dialysis, medication, fluid and dietary recommendation were 8.5%, 49.5, 51.6% and 63.8%, respectively (Table 3). According to the degree of deviation, majority of the subjects deviated either mildly or moderately from the recommended regimens. There were however a total of 19.7% and 11.7% of the subjects reported severe and very severe degrees of deviation from dietary and medication recommendation, respectively.

**Table 2:** Comparison between clinically determined and self-reported compliance rates.

Compliance Indicator	Clinically Determined compliance rates (%)	Self-reported compliance rates (%)
Dietary	27.7^1^	36.2
Fluid	24.5^2^	48.4
Medication	66.5^3^	50.5
Attendance to dialysis	91.0^4^	91.5

1 Both serum potassium and phosphorus achieved compliance criteria.

2 IDWG achieved compliance criteria.

3 Serum phosphorus achieved compliance criteria.

4 17 subjects skipped at least one dialysis session (data derived from dialysis record).

5 16 patients self-reported missing at least one dialysis session.

**Table 3:** Self-reported of intensity of treatment compliance.

Deviation of Regimen		Degree of deviation	Mean Compliance Score	Frequency	%
Dietary			2.73		
	No deviation			68	36.2
	With deviation			120	63.8
		Mild		50	26.6
		Moderate		33	17.5
		Severe		25	13.3
		Very Severe		12	6.4
Fluid*			3.06		
	No deviation			90	48.4
	With deviation			96	51.6
		Mild		34	18.3
		Moderate		50	26.9
		Severe		8	4.3
		Very Severe		4	2.1
Medication			3.03		
	No deviation			95	50.5
	With deviation			93	49.5
		Mild		34	18.1
		Moderate		37	19.7
		Severe		14	7.4
		Very Severe		8	4.3
Dialysis			3.91		
	No deviation			172	91.5
	With deviation			16	8.5
		Mild		16	8.5
		Moderate		0	0
		Severe		0	0
		Very Severe		0	0

* Two subjects refused to disclosure information.

Perceived barriers contributing to non-complaint to treatment regimens were identified and are shown in Table 4. A total of 86.2% of the subjects admitted compliant to fluid prescription was the most difficult and challenging aspect, especially during hot weather while 72.9% reported difficulty following their dietary prescription. This was followed by 52.1% who reported had difficulty taking medications as prescribed. The need to change eating habits and inability to resist favourite foods (88.1%) and the highly complexity of dietary recommendation (87.0%) were the major factors cited for dietary non-compliance, superimposed the knowledge factor (74.7%). On the other hand, lacking of knowledge or information pertaining to fluid management was the major factor cited for fluid non-compliance (92.8%) followed by the complexity of fluid management. Forgetfulness, associated side effects/complications and complexity of the prescribed medications treatment were the three major factors perceived by patients contributing to non-compliance to medications. A majority (70.6%) of the subjects reported they had difficulty adhering to phosphate binder *per se* due to its associated side effects such as constipation and the unpleasant experience to take large quantities with meals. Large tablet burden was reported as barrier to compliance for 60.6% of the subjects. There were 12.2% of the subjects admitted having difficulty to comply with dialysis attendance attributed by financial constraint and lacks of transportation facility.

As shown in Table 5, there were positive correlations between age and compliance on dietary (*r* = 0.186, *p*<0.05), fluid (*r* = 0.385, *p*<0.01) and medication (*r* = 0.271, *p*<0.01), respectively, indicating younger subjects were more non-compliant to the therapeutic regimen compared to their older age counterparts. Female subjects were statistically more compliant to dietary (*r* = 0.252, *p*<0.05) and fluid restriction (*r* = 0.310, *p*<0.01). There was however no significant different between male and female subjects on medication compliance (*r* = 0.172, *p*>0.05). Employment status was found to be inversely related to dietary (*r* = −0.355, *p<*0.01) and fluid (*r* = −0.441, *p*<0.01) compliances. These results suggested that subjects who were employed were more likely to be non-compliant to dietary and fluid restrictions. Longer hemodialysis vintage was associated with poorer compliances on fluid (*r* = −0.410, *p<*0.01) and medication (*r* = −0.368, *p*<0.01), implying that patients who had longer lengths of time on dialysis were more likely to have hyperphosphatemia and gained more IDWG. Knowledge scores on potassium and phosphorus were negatively correlated with compliance on dietary (*r* = −0.345, *p*<0.01) and medication (*r* = −0.278, *p*<0.05), respectively. On the other hand, there were no significant correlations between knowledge scores on fluid or sodium with dietary, fluid or medication compliance. These findings suggest that higher knowledge on dietary aspects may not associated with better compliance rates.

**Table 4:** Perceived barriers contributing to non-compliance on treatment regimens.

		Dietary (%)	Fluid (%)	Medication (%)	Dialysis Attendance (%)
Non-compliance Rate^1^		63.8	51.6	49.5	8.5
Difficult to comply 2		72.9	86.2	52.1	12.2
Perceived barriers	Lacks of knowledge or information^3^	74.7	92.8	55.4	NA
	Affects food preference	88.1	64.2	36.1	NA
	Alters lifestyle^4^	69.2	56.8	54.7	NA
	Complexity^5^	87.0	75.4	75.8	NA
	Side effects/Complications	NA	NA	78.3	NA
	Forgetfulness	NA	NA	80.6	NA
	Large tablet burden	NA	NA	60.6	NA
	Financial constraint and lacks of transportation facility	NA	NA	NA	100

1 Data derived from Table 2.

2 Percentage of subjects who admitted having difficulty adhering to the treatment regimens (multiple responses were possible).

3 Lacks of knowledge (e.g. water allowance for different weather conditions, type of foods to be consumed or restricted).

4 Alters lifestyle (e.g. the need to consume mega dosage of supplements and medicines).

5 Complexity (e.g. Nutrient composition of different foods; when and how to consume phosphate binders).

Self-reported dietary compliance score was positively correlated with compliance on dietary (*r* = 0.236, *p*<0.05) and medication (*r* = 0.197, *p*<0.05), while self-reported medication compliance score was correlated with compliance on medication (*r* = 0.412, *p*<0.01). There were also significant correlations between fluid compliance with self-reported compliance score on fluid (*r* = 0.342, *p*<0.05) and dialysis (*r* = 0.213, *p*<0.05). These data may suggest that the self-reported data can be used to determine the compliance behaviors of hemodialysis patients, in the absence of clinical measures. On the other hand, there were no significant associations between compliance indicators and education level or family income.

**Table 5:** Correlation between compliance behaviors and variables.

Variables		Dietary Compliance	Fluid Compliance	Medication Compliance
Age		0.186*	0.385**	0.271**
Sex^1^		0.252*	0.310**	0.172
Education level		−0.124	−0.102	−0.108
Employment Status		−0.355**	−0.441**	−0.187
Family income		−0.129	−0.138	−0.115
Vintage on hemodialysis		−0.152	−0.410**	−0.368**
Knowledge scores on	Potassium	−0.345**	0.167	0.109
	Phosphorus	0.162	0.134	−0.278*
	Fluid	0.087	0.123	−0.153
	Sodium	0.113	−0.162	0.144
Self-reported Compliance score	Dietary	0.236*	0.166	0.197*
	Fluid	−0.174	0.342*	−0.156
	Medication	−0.151	−0.152	0.412**
	Dialysis	0.137	0.213*	0.102

1 Female is a reference group in sex.

* p<0.05.

** p<0.01.

Stepwise multivariate linear regression analyses were performed to identify variables that predict the compliance behaviours. As displayed in Table 6, higher fluid compliant was predicted by female gender (*β* = 0.207, *p*<0.05), older age (*β* = 0.195, *p*<0.05), higher self-reported fluid compliance score (*β* = 0.168, *p*<0.05), shorterhemodialysis vintage (*β* = −0.155, *p*<0.01) and being not employed (*β* = −0.125, *p*<0.05). Meanwhile, higher dietary compliance was predicted by higher self-reported dietary compliance score (*β* = 0.250, *p*<0.05), female gender (*β* = 0.162, *p*<0.05), older age (*β* = 0.147, *p*<0.05) and no employment (*β* = −0.142, *p<*0.05) while higher medication compliance was significantly predicted by higher self-reported medications compliance score (*β* = 0.353, *p*<0.01), shorter hemodialysis vintage (*β* = −0.224, *p*<0.05) and older age (*β* = 0.181, *p*<0.05). The variation for these three models ranged from 21.5–39.2%.

**Table 6:** Standardized coefficients of the linear regression model predicting compliance.

Compliance Behaviors	Variables	Standardized coefficients (β)	Adjusted R^2^
Fluid	Age	0.207	0.392
	Sex^1^	0.195	
	Fluid compliance score	0.168	
	Hemodialysis vintage	−0.155	
	Employment^2^	−0.125	
Dietary	Dietary compliance score	0.250	0.341
	Age	0.162	
	Sex^1^	0.147	
	Employment^2^	−0.142	
Medication	Medications compliance score	0.353	0.215
	Hemodialysis vintage	−0.224	
	Age	0.181	

1 Female is a reference group in sex.

2 Job engagement is a reference group in employment.

## Discussion

Diabetes mellitus accounted for the primary renal disease in this cohort of study, which is also a characteristic reported by the national renal registry of Malaysia [1]. A high proportion of hemodialysis patients in Malaysia had difficulty in following diet and fluid restriction. This finding is consistent with the range of compliance behaviours reported in other studies among dialysis patients [12]–[13], [28]. Compared to a recent study conducted in Hong Kong [26], our compliance rates on dietary and dialysis were comparable, but the compliance rates on fluid and medication were 24% and 39% lower. We compared with another study in United States [24] and found that their compliance rates of diet, fluid and medication were 26–47% higher than those reported in our study. A recent publication on the diabetes control also revealed that there was poor compliance on diet, exercise and self-monitoring blood glucose among type 2 diabetics in Malaysia [32]. Although evidence is still lacking to generalise whether Malaysians are less likely to adhere to medical regimes than other populations, the available data suggest that extra efforts and appropriate strategies are needed to assist our hemodialysis patients to achieve the desirable recommendations, especially on fluid and medication.

The findings that our subjects perceived themselves as more compliant to dialysis than medication prescription, dietary or fluid restrictions are similar to earlier studies [24], [26], [33]. This may be attributed by the need for higher willpower, more appropriate knowledge and skill to achieve dietary and fluid recommendations. The mean self-reported compliance rates for fluid and dietary were 9% and 19% higher than the clinically determined rates. Other studies have also reported that hemodialysis patients consistently overestimated their compliance to fluid and dietary recommendations [13], [27], [34]. There is no clear explanation for this, but it is likely that the long duration of dependence on dialysis (length of time on dialysis) may cause hemodialysis patients to accustom to the restrictions imposed by the disease and perceived themselves as having better compliance than they actually did. Secondly, the use of clinical data for example serum potassium and phosphorus as the direct measures of dietary compliance could be misleading as these clinical data may also be affected by factors such as dialysis adequacy, medication and other factors yet to be identified. On the other hand, self-reported medication compliance was found to be underestimated by 16% as compared to clinically measured compliance indicator. Tomasello et al. (2004) [35] reported a similar finding where non-compliance to treatment was 58% and 31% when assessed using clinical and self-report measures, respectively. This may provide an impetus that using a single indicator to document the overall medication compliance rate could be insufficient and thus more comprehensive assessment tool is therefore needed.

Older age appeared to be the most important predictor among all predictor variables, explaining variance in all three compliance behaviours. Other studies have also reported that older age was associated with higher compliances to fluid restriction and medication prescription [24], [36]–[37]. Possible explanations are older patients may have more structured lifestyle that accommodates the demands of the treatment regimen while younger patients may perceive themselves as less vulnerable to negative health outcomes [38], confirming the existence of an “intentional noncompliance” [39]. The finding that younger patients were more likely to be non-compliant to treatment recommendation may lead to the future poorer quality of life and higher rates of mortality among these dialysis patients. The dialysis patients in Malaysia are perceived as relatively young [40]. In view of the younger cohort of dialysis patients together with the higher tendency of these patients to be non-compliant to treatment, it is highly recommended that action plans need to be formulated to address the projected higher mortality rates and poorer quality of life among our dialysis population. We shared that women were more compliant to diet and fluid restriction than men and this findings are similar to other studies [12], [26], [31]. Female hemodialsysis patients had consistently reported to have a lower adjusted hazard ratio for mortality compared to their male counterparts in Malaysia [1]. It is likely that women are more health conscious than men. How gender differences in compliance may benefit patients concerning health outcomes in the long run however deserves for longitudinal research.

The correlations between higher self-reported compliance to medication and dietary compliance with lower phosphorus levels are of particular importance. Cardiovascular events are the leading cause of death in dialysis patients. The increased incidence of cardiovascular event in dialysis patients is associated with hyperphosphatemia [30], making phosphate control an important goal of treatment. While dialysis *per se* cannot remove the significant quantities of phosphate from the body, the appropriate restriction in dietary phosphorus intake and use of phosphate binder are therefore critically important to manage hyperphosphatemia. It is then necessary for hemodialysis patients to comply with *both* the dietary phosphorus intake and phosphate binding medication in reducing the risk of adverse clinical outcomes.

We demonstrated that higher knowledge scores were not associated with better compliance rates, which suggest that knowledge is not the sole factor related to compliance rate. This finding is congruent with other studies [11]–[12], [37]. While Zrinyi et al. (2003) [23] showed that employment may improve the dietary compliance and it is associated with better patient-staff relationships, we found that hemodialysis patients who were employed were more likely to be non-compliant to diet and fluid restriction. This concurs with other study [13]. Working dialysis subjects may consume more outside foods that contain generally higher amount of sodium and potassium, which could lead to a higher challenge in handling thirst stimulus and subsequent increased in serum potassium level.

We found that subjects with longer duration on hemodialysis were more non-compliant. This finding concurs with other studies [13], [25], [41]. It is postulated that end stage renal disease patients may be more eager to change their dietary habits to meet the requirement of a newly-received life-saving hemodialysis treatment. However as time passes, these patients may feel bored and easily get frustrated with the need to comply with long lists of dietary and fluid restrictions [26]. Patients new to dialysis treatment may also receive more social support and were therefore higher degree of compliant is expected [26]. However, over the long run, it may be difficult for patients to resist the wide variety of food available. In view of this, healthcare providers should identified the individual's perceived barrier, explore patients' willingness and readiness to make changes to their dietary habits to achieve the optimum effect of compliance.

In conclusion, the majority of our subjects were compliant to dialysis prescription. However, compliance to other regimens especially for fluid and diet restriction remains poor. Younger male, working patients and those with long experience with dialysis warrant increased scrutiny and deserve for special attention and support. Besides knowledge, lacking of appropriate self-efficacy skills and regimen complexity were the perceived barriers hindering better compliance from the patients' perspective. Healthcare professionals should recognize that the perceived barriers to compliance vary according to types of treatment. For example, while adequate knowledge and information are needed to improve fluid compliance, appropriate self-efficacy skills and coping strategies are needed to enable hemodialysis patients to achieve the optimum effect of dietary compliance. Messages deliver by the healthcare professional should be “simple and practical”, allowing the patients to understand and practice the messages within their capabilities. Reinforcement of messages together with more frequent counseling encounters may promote a better understanding of the prescriptions subsequently a higher compliance rates among the patients.

Our study design was cross-sectional in nature and the sample size was relatively small which could limit the cause-effect interpretation and generalisation of finding. Selection bias is also possible where only patients who were generally healthier or more health conscious were more likely to participate in the study. Despite these limitations, the study highlighted several important findings that require further investigations using stronger research design and larger sample size.
